# Advances in percutaneous coronary intervention for chronic total occlusions: current antegrade dissection and reentry techniques and updated algorithm

**DOI:** 10.1007/s12471-020-01509-8

**Published:** 2020-11-06

**Authors:** T. Berkhout, B. E. Claessen, M. T. Dirksen

**Affiliations:** Department of Cardiology, Noordwest Ziekenhuisgroep, Alkmaar, The Netherlands

**Keywords:** Humans, Coronary angiography, Coronary occlusions, therapy, Percutaneous coronary intervention, methods, Decision support techniques, Clinical competence

## Abstract

Percutaneous coronary intervention (PCI) for chronic total occlusions (CTO) is considered relatively complex with low success rates and high complication rates. Treating a CTO with PCI using the hybrid algorithm increases success rates with acceptable complication rates. An essential part of the hybrid algorithm is antegrade dissection and reentry (ADR). In PCI of a non-CTO coronary lesion, the guidewire over which the stent is advanced and placed stays within the true lumen of the coronary artery. ADR techniques make it possible to cross the lesion through the wall of the coronary artery, the subintimal space, thus creating a small bypass within the architecture of the coronary artery and restoring antegrade blood flow. ADR increases success rates, especially in more difficult CTO procedures. In the last decade, new materials and techniques have been introduced in quick succession, which are summarised in this review. Consequently an updated ADR algorithm is presented, which can support the CTO operator during an ADR procedure.

## Introduction

A coronary chronic total occlusion (CTO) is defined as the absence of antegrade transluminal flow (thrombolysis in myocardial infarction (TIMI) grade 0 flow) for an estimated duration of more than 3 months. Compared to non-CTO coronary lesions, CTOs are considered relatively complex, with lower success rates and higher complication rates [1]. It is known that patients with CTO have higher mortality than patients without CTO [2]. In addition to increased prevalence of comorbidities, potential explanations for the finding of increased mortality include an increased incidence of ventricular arrhythmia [3] and ‘double jeopardy’ if patients suffer a myocardial infarction, as the infarct-related artery may also supply collaterals to the CTO, resulting in a larger area of subtended myocardium. The threshold for revascularisation of CTO is higher than for non-CTO lesions, but percutaneous coronary intervention (PCI) of a CTO should be considered when a patient has symptoms, documented ischaemia or left ventricular dysfunction [4, 5].

In recent years, the field of CTO-PCI has evolved considerably as many new materials and techniques have been introduced. This article aims to provide an overview of the most important contemporary antegrade dissection and reentry (ADR) techniques, where the CTO is crossed through the subintimal space (SIS) instead of the intraluminal route. Moreover, we propose an updated algorithm to guide the interventional cardiologist in performing CTO-PCI.

## Modern PCI for CTO

Historically, CTO-PCI success rates have been low, typically 70–80% [6]. Algorithms were developed, such as the hybrid approach [7] and the Asia Pacific algorithm [8], which increased success rates. These algorithms aid in teaching the knowledge needed to treat patients with CTO. The hybrid algorithm recommends a standardised approach for evaluating the CTO, to determine the initial approach (antegrade or retrograde) and offers guidance on when to switch between CTO techniques [9]. The hybrid algorithm, along with the introduction of high-volume CTO programmes with dedicated CTO operators, resulted in higher success rates and acceptable complication rates [6].

The hybrid algorithm includes two techniques, wire escalation and dissection and reentry techniques (DART), which can be used in antegrade or retrograde fashion. In wire escalation, interventional guidewires are used to cross the CTO body rapidly, progressing from low-tip-load guidewires to higher-tip-load guidewires or guidewires with different coatings. The aim of this technique is to cross the CTO body without leaving the lumen.

When using DART, the entire vessel architecture is used to cross the CTO body. The SIS is entered in front of the CTO body, either with a guidewire or dedicated material. The CTO body is then crossed through the SIS. Distal to the CTO body the guidewire is reentered into the true lumen.

**Fig. 1:**
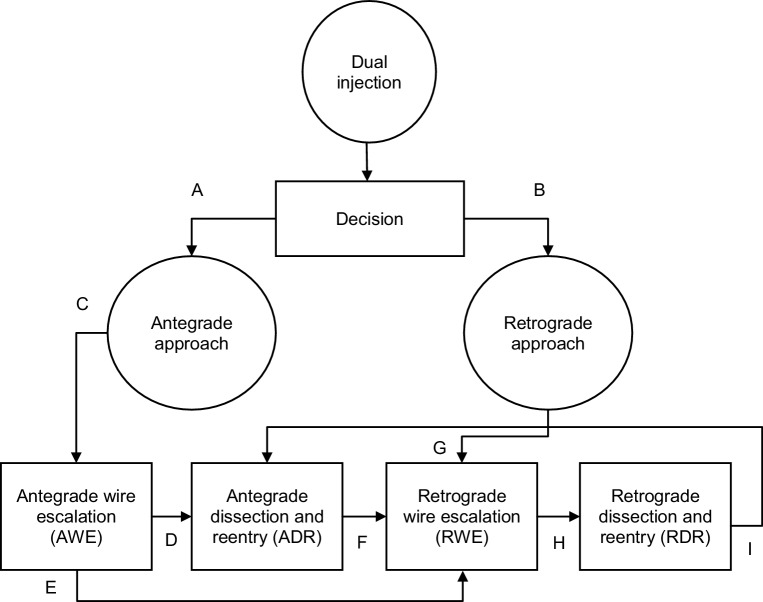
Hybrid approach algorithm for percutaneous coronary intervention (*PCI*) for chronic total occlusions. *A*. If proximal cap clearly defined, available landing zone with no major bifurcation; *B*. If ambiguous proximal cap, ostial lesion, disease or bifurcation in landing zone, available interventional collaterals; *C*. First antegrade wiring unless lesions longer than 20 mm; *D*. If AWE unsuccessful and available landing zone start ADR; *E*. If AWE unsuccessful and available interventional collaterals start RWE; *F*. If ADR unsuccessful switch to RWE if interventional collaterals available. Otherwise modify antegrade cap, abort intervention and arrange for repeat PCI; *G*. Start retrograde wiring of collaterals and CTO body if ambiguous proximal cap, bifurcation in landing zone and available collaterals; *H*. Switch to RDR if no progress made with RWE; *I*. If not yet tried, switch to ADR

These techniques combined provide four options as can be seen in Fig. 1: (1) antegrade wire escalation (AWE), (2) ADR, (3) retrograde wire escalation (RWE) and (4) retrograde dissection and reentry (RDR).

The ADR technique is recommended if the CTO body has of one or more of the following anatomical features: (1) tapered cap, (2) length >20 mm, (3) large-diameter distal vessel and (4) no large side branches near the distal cap [10]. If these features are not present in the anatomy of the coronary arteries and interventional collaterals are available, a retrograde approach is preferred. ADR is used in about 20–25% of all CTO-PCI cases [11]—in 30% as the initial technique and in 70% after an initial AWE or RWE/RDR attempt. The need for ADR rises dramatically in cases with a high Japanese CTO Registry (J-CTO) score, which specifies the expected difficulty and success rates of the CTO-PCI based on the pathophysiology of the CTO and surrounding coronary artery [12, 13]. When ADR is applied a success rate of 66% is reported [11]. If wiring the CTO is unsuccessful while using the ADR technique and no retrograde options are available, subintimal plaque modification (SPM) can be applied, which alters the CTO body, for example with balloon dilatation. A repeat PCI can be scheduled for a second attempt with possibly favourable changes in the CTO body. The ADR technique comprises three distinct stages. First, the SIS must be entered, then the CTO body must be crossed, and finally the distal vessel needs to be reentered.

### Making a dissection and entering the SIS

When ADR techniques are likely to be applied, trapping devices, such as a TrapLiner catheter (Teleflex, Wayne, PA, USA) or a Trapit balloon catheter (IMDS, Roden, The Netherlands), can be useful for fixating the guidewire with a balloon in the guiding catheter for exchanging, for example, microcatheters while maintaining the most distal position of the guidewire. Most primary antegrade procedures start with an AWE attempt. If the guidewire enters the SIS, the parallel wiring technique can be attempted. The guidewire in the SIS can sometimes redirect a second guidewire towards the true lumen. This can be useful in cases with (1) no calcification in the distal cap, (2) first guidewire in the CTO body, (3) large-diameter distal vessel, (4) good retrograde visualisation and (5) expected ease of delivery of a dual lumen catheter [14]. If this fails or is not an option, a decision needs to be made as to whether to continue via the antegrade route or to switch to retrograde options, if available.

Before continuing with an ADR strategy, it is important to verify this with a retrograde contrast injection to assess if the guidewire has left the lumen but is still moving in phase with the treated coronary artery, indicating a subintimal position. Otherwise it is possible that the guidewire has perforated. Antegrade contrast injections should be avoided because of the risk of hydraulic dissections. Making a selective injection via ipsilateral collaterals through a microcatheter can be considered in order to establish the position of the guidewire.

Several techniques have been reported with which to enter the SIS in situations where a dissection is difficult. Nonetheless, this step of ADR remains a frequent reason for PCI failure. The techniques are: (1) scratch and go, (2) balloon-assisted subintimal entry (BASE), (3) Carlino and (4) grenadoplasty.

To perform the scratch and go technique, a microcatheter is used in combination with a high-tip-load guidewire, which can make a small puncture in the intima via which the microcatheter is advanced. The high-tip-load guidewire is replaced with a polymer-coated, low-tip-load guidewire and is pushed forward, forming a large J‑tip in the guidewire (knuckle). This creates a dissection plane, while the large knuckle prevents the guidewire from entering small side branches. The disadvantage of using a knuckle is the larger dissection plane, which makes reentering the true lumen difficult later in the process because of lumen compression owing to antegrade blood flow into the SIS [4]. Different guidewires can be used to form a knuckle, such as the XT‑A (Asahi Intecc, Akatsukicho, Japan) or pilot 200 (Abbott, Lake Bluff, IL, USA). A newer, dedicated guidewire for knuckling, such as the Gladius MG PV (Asahi) can be used, as it forms a smaller and more controlled knuckle, which limits the forming of a large SIS.

In the BASE technique, a balloon is inflated in front of the CTO body to modify the architecture of atherosclerosis and to create a dissection in the intima [15]. After balloon inflation a polymer-coated, low-tip-load guidewire is advanced into the SIS. This can cause distal lumen compression, which complicates distal reentry.

A modification of this technique is the side-BASE [16], which can be useful when dealing with a blunt, ambiguous cap of the CTO body with a side branch next to the cap. A balloon is inflated in the side branch, and this redirects the guidewire to the cap. A polymer-coated guidewire can be used to start a dissection.

The Carlino technique is another modification of the scratch and go strategy, also using a microcatheter and high-tip-load guidewire to make a puncture [17]. To create a dissection, an injection of contrast is given. As the contrast fills the SIS, three scenarios can occur:

1. No contrast enters the SIS and the process can be repeated.
2. A tubular dissection forms in the SIS. This helps with steering the guidewire towards the true lumen.
3. A ‘storm cloud’ dissection is observed. This often preludes failure of the CTO-PCI, and the operator should closely monitor the patient for the possibility of cardiac tamponade.

Grenadoplasty can be used to alter the architecture of the CTO [4]. Depending on the estimated vessel diameter, a 1.2- to 1.5-mm balloon is advanced into the CTO; then the balloon is inflated until it bursts. This creates a dissection and changes the composition of the proximal cap of the CTO. This can be helpful in entering the SIS.

### Crossing the CTO body

**Fig. 2:**
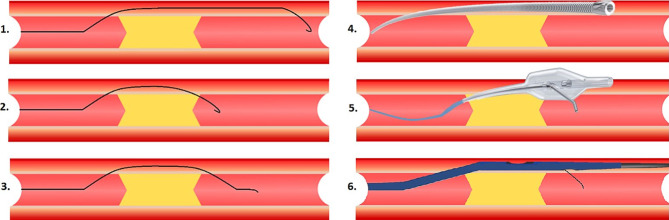
*1* STAR technique, *2* mini-STAR technique, *3* LAST technique (wire-based techniques) and *4* CrossBoss, *5* Stingray balloon, *6* ReCross

During an ADR procedure it is key to limit the size of the SIS and prevent excessive haematoma formation, compressing the true lumen. In so doing, the distal true lumen remains large, making reentry easier and helping to visualise the distal vessel and verify a successful reentry. Crossing the CTO body through the SIS is an important addition to CTO-PCI. Transluminal traversing of the CTO body results in CTO-PCI with low success rates and a higher risk for perforation, especially in long, calcified or tortuous vessels [18]. Wiring the CTO through the SIS makes this process easier and safer, but does introduce the necessity to make a (1) wire-based or (2) device-based reentry distal to the CTO body [6]. Fig. 2 shows the mostly used techniques. Excellent schematic representations of the different techniques can also be found in different CTO manuals [4, 19, 20].

#### Wire-based techniques

Once in the SIS, a guidewire can be used to create a dissection plane next to the CTO body. Wire-based ADR techniques are considered unpredictable and lacking control [10]. Several techniques are used as bail-out strategies in clinical practice:

1. Subintimal tracking and reentry (STAR) technique, mini-STAR technique and contrast-assisted STAR technique [21]
2. Limited antegrade subintimal tracking (LAST) technique [4]
3. Antegrade fenestration and reentry (AFR) technique [22]

The ‘subintimal tracking and reentry’ (STAR) technique (Fig. 2, part 1) was first used in peripheral interventions before being introduced for coronary interventions [23]. A microcatheter is used in combination with a polymer-coated knuckled guidewire. The knuckled guidewire is advanced until reentry into the distal vessel is achieved, often after a bifurcation. This technique can be useful in CTO in the right coronary artery, but not in the left anterior descending artery because of side branch loss.

The mini-STAR technique (Fig. 2, part 2) is used in an attempt to limit the length of the dissection plane by making a more proximal dissection. This is achieved by using a polymer-coated guidewire with a 45-degree angle at the tip and a second 15-degree angle 4 mm from the tip. Two scenarios are possible: (1) the guidewire follows a microchannel through the CTO to the distal true lumen (AWE) or (2) a knuckle is formed in the SIS which can reenter into the distal true lumen [24].

The contrast-assisted STAR technique is usually performed in accordance with the previously described Carlino technique [25], whereby the SIS is visualised by injection of contrast, which helps the operator in wiring the SIS and achieving a reentry. This technique has largely been abandoned due to improved visualisation of the proximal and distal parts of the CTO by using dual injections.

Although an innovative technique at the time, the STAR technique has low success rates and creates large dissection planes, often results in loss of side branches, and has a high target lesion revascularisation rate (±50%) [21]. It is now used only as a bail-out technique or as a so-called SPM. After a few weeks/months coronary flow and visualisation will improve due to resorption of haematoma, resulting is higher success rates, limiting stent length and preventing side branch occlusions [24].

The limited antegrade subintimal tracking (LAST) technique (Fig. 2, part 3) involves creating a small dissection plane with a more proximal and deliberate reentry than in the STAR technique [9]. Instead of using a polymer-coated low-tip-load guidewire, a high-tip-load guidewire is used.

Another technique for reentering the true lumen is AFR [22]. After attaining a subintimal position behind the distal cap a second guidewire is positioned close to the first guidewire. After verifying that the position of both guidewires is roughly in the same part of the SIS, a balloon is advanced, sized according to the original size of the coronary artery. Then the balloon is inflated in the SIS with the aim of fenestrating the intima, thus creating a route for the first guidewire to enter the true lumen.

Wire-based ADR techniques show lower success rates compared to device-based techniques [26]. Moreover, improvement in myocardial blood flow after CTO-PCI was reported to be lower with wire-based than with device-based reentry techniques [27].

#### Dedicated devices for reentry

The Stingray balloon (Boston Scientific, Marlborough, MA, USA) is the most frequently used device for ADR, often preceded by traversing the SIS with the CrossBoss (Boston Scientific) or microcatheter (Fig. 2, parts 4 and 5). The CrossBoss catheter is a blunt dissection tool with a 1-mm rounded tip, which is rapidly rotated by the operator while exerting forward pressure. In comparison with a knuckled guidewire the main advantage is a controlled, limited dissection which facilitates a reentry into the true lumen distal to the CTO. In a minority of cases it crosses the CTO intraluminally. In some cases, the CrossBoss does not pass the first section into the CTO or SIS, in which case a high-tip-load guidewire can be used to advance the CrossBoss the first few millimetres. If progress is stalled in the CTO body or the CrossBoss enters side branches, a knuckled polymer-coated guidewire can be used to cross the largest part of the CTO, switching back to the CrossBoss to prepare the landing zone in the distal vessel [10]. In occluded stents, the CrossBoss can used, especially when: (1) the CTO has a clear proximal cap, (2) the distal cap is in the stent and (3) the stent has been properly expanded [14]. For CTO-PCI in which ADR techniques are a probable option, 7 French (or larger) guide catheters are needed to handle the CrossBoss and Stingray balloon.

An alternative is the ‘bougie technique’, where a Stingray balloon can be used without using the CrossBoss to create a SIS [14]. If a guidewire has entered the SIS and crossed to the distal landing zone a microcatheter can be used to predilate the subintimal track. Then a Stingray balloon should be advanced to subsequently perform a reentry into the true lumen. If the guidewire spirals around the vessel it can be hard to deliver the Stingray balloon. The use of the CrossBoss could overcome this by creating a straight track. Distal lumen compression can hinder reentering the true lumen and should be prevented. This is done by limiting the SIS, preventing blood flow by inflating a balloon proximal to the CTO, or by advancement of a guide extension.

**Fig. 3:**
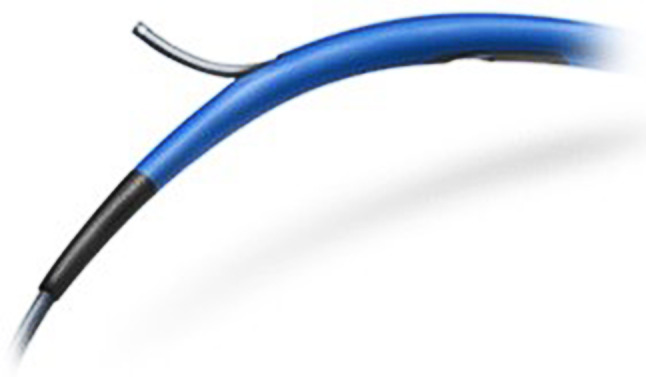
ReCross Dual Lumen OTW Microcatheter (Interventional Medical Device Solutions)

A new, less well established reentry device is the ReCross Dual Lumen OTW Microcatheter (IMDS) (Fig. 2, part 6; Fig. 3). This is a hydrophilic coated dual lumen catheter with a distal oval shaft. The ReCross also has a removable stylet which increases pushability, facilitating passage beyond the CTO body. This all-in-one device was developed for crossing the CTO body as well as reentering the true lumen. It creates a smaller SIS, compared to the CrossBoss. This microcatheter has two exit ports which can be used for making a reentry. If reentering the true distal lumen is unsuccessful the ReCross can be replaced with a Stingray balloon for a more controlled attempt.

### Device-based reentry of the true lumen

The first step in reentering is selecting a suitable landing zone. This decision can be based upon the following factors: (1) calcification, (2) plaque burden, (3) diameter, (4) length, (5) severity of haematoma, (6) tortuosity of landing zone and (7) proximity to large side branch and (8) experience of operator [14].

Wire-based reentry techniques have been described above. After a dissection is made with the CrossBoss or microcatheter, it is removed while a guidewire remains within the SIS. While using an exchange guidewire or a trapping device, the CrossBoss is replaced with a Stingray balloon. The Stingray balloon catheter is an over-the-wire 2.5-mm balloon catheter with an exit port facing towards the vessel and one facing the adventitia. A third port is located on the tip and can be used for subintimal transcatheter withdrawal (STRAW), a technique for removing blood from the SIS and reducing distal vessel compression [10]. A disadvantage can be reduced wire control because of blood in the lumen of the Stingray balloon. The STRAW technique can also be used with other devices, such as the ReCross. When inflated, the Stingray balloon self-orientates on top of the vessel. The operator has to assess the position of the radio-opaque markers to confirm that the balloon is well positioned. Next a high-tip-load guidewire is advanced to puncture the intima to access the true lumen. Successful reentry can be verified by a retrograde contrast injection.

**Fig. 4:**
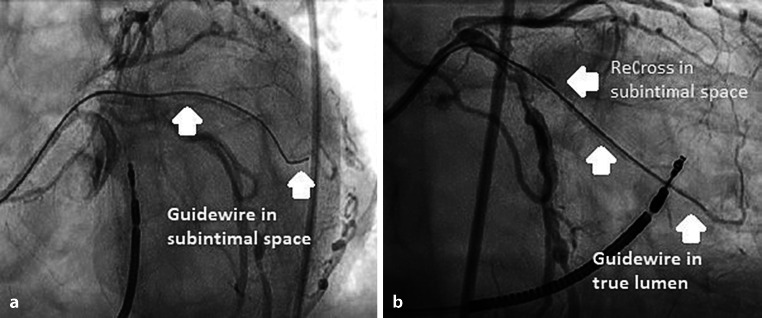
**a** The guidewire is in a subintimal position in the left marginal artery. **b** The ReCross can be seen in the subintimal space and the guidewire in the true lumen

The ReCross has two exit ports on the side of the device, oriented at 180 degrees from each other (and an additional one at the distal tip end), but lacks the radio-opaque markers for orientation in the vessel. This makes a controlled reentry more difficult. However, in a distal vessel with a larger lumen this device can facilitate fast dissection and reentry. In Fig. 4a panel, a guidewire can be seen in the SIS. In Fig. 4b panel, the ReCross can be seen proximally in the obtuse marginal branch, and in the distal vessel the guidewire is in the true lumen. The advantages of the ReCross are that it is fast, cheap and simple to operate in comparison to the CrossBoss and Stingray devices. The disadvantages are that it can be difficult to observe the orientation of the device in relation to the distal target. The overall success rates require further study.

To reenter the true lumen different techniques can be used: (1) stick and drive and (2) (multi) stick and swap approach. In stick and drive manoeuvres a high-tip-load guidewire is used to puncture the vessel wall and is then driven into the true lumen. The stick and swap approach is performed using a high-tip-load guidewire to make a puncture and is then swapped for a polymer-coated guidewire to wire the distal vessel. In the multi-stick approach several sticks are made to create a larger puncture, from where the polymer-coated guidewire tracks better to the true lumen.

The position of the guidewire in the true lumen can be assessed using a retrograde contrast injection. It can be helpful to move the guidewire during contrast injection to see if the guidewire follows the true lumen and enters side branches.

## Updated ADR algorithm

**Fig. 5:**
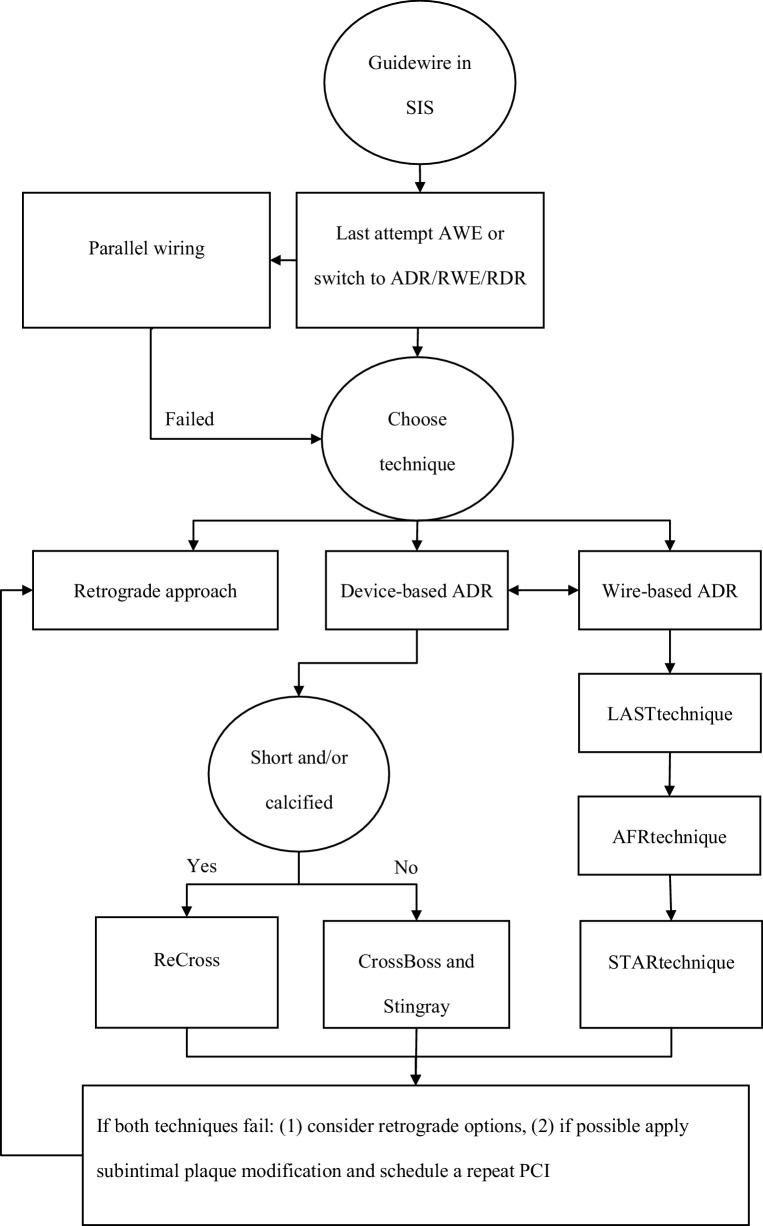
Updated algorithm for antegrade dissection and reentry. *SIS* subintimal space, *AWE* antegrade wire escalation, *RWE* retrograde wire escalation, *RDR* retrograde dissection and reentry, *LAST* limited antegrade subintimal tracking, *AFR* antegrade fenestration and reentry, *STAR* subintimal tracking and reentry, *PCI* percutaneous coronary intervention

The previously described materials, techniques and strategies can be combined in an algorithm which includes all important contemporary steps of ADR (Fig. 5). Most primary antegrade procedures start with an AWE attempt. A result of AWE can be that the guidewire exits the lumen and enters the SIS. This opportunity can be used for ADR. The preferred method of ADR includes using a dedicated device such as the CrossBoss/Stingray or possibly the ReCross catheter. By using these devices it is possible to make a safe and controlled reentry. If the CTO is short and/or calcified the preferred choice could be the ReCross catheter and in longer and/or calcified cases the CrossBoss/Stingray combination should be used. After an unsuccessful ReCross attempt, switching to the Stingray balloon could be considered to make a more controlled reentry. If the device-based approach fails, wire-based techniques can be attempted. However, this is unlikely to prove successful after a device-based strategy has failed.

If all ADR techniques fail, switching to a retrograde option can be considered or SPM followed by a staged procedure. The success rates of a staged procedure after SPM seem reasonable [6].

## Conclusions

The materials and techniques for PCI for CTO have developed substantially since the introduction of the hybrid approach, resulting in increased success rates. In this article we describe these advances in ADR and propose an updated algorithm which can help the interventional cardiologist and nursing staff in treating patients during CTO-PCI.

## References

[1] Riley RF, Sapontis J, Kirtane AJ (2018). Prevalence, predictors, and health status implications of periprocedural complications during coronary chronic total occlusion angioplasty. EuroIntervention.

[2] Råmunddal T, Hoebers L, Henriques JPS (2014). Chronic total occlusions in Sweden—a report from the Swedish Coronary Angiography and Angioplasty Registry (SCAAR). PLoS One.

[3] Chi WK, Gong M, Bazoukis G (2018). Impact of coronary artery chronic total occlusion on arrhythmic and mortality outcomes: a systematic review and meta-analysis. JACC Clin Electrophysiol.

[4] Brilakis E (2018). Manual of chronic total occlusion interventions, a step-by-step approach.

[5] Sousa-Uva M, Neumann FJ, Ahlsson A (2019). 2018 ESC/EACTS guidelines on myocardial revascularization. Eur Heart J.

[6] Wilson WM, Walsh SJ, Yan AT (2016). Hybrid approach improves succes of chronic total occlusion angioplasty. Heart.

[7] Brilakis ES, Grantham JA, Rinfret S (2012). A percutaneous treatment algorithm for crossing coronary chronic total occlusions a percutaneous treatment algorithm for crossing coronary. JACC Cardiovasc Interv.

[8] Harding SA, Wu EB, Lo S (2017). A new algorithm for crossing chronic total occlusions from the Asia Pacific Chronic Total Occlusion Club. JACC Cardiovasc Interv.

[9] Micheal T, Mogabgab O, Fuh E (2014). Application of the “hybrid approach” to chronic total occlusion interventions: a detailed procedural analysis. J Interv Cardiol.

[10] Wyman RM (2012). Antegrade dissection and reentry: tools and techniques. Interv Cardiol Clin.

[11] Maeremans J, Dens J, Spratt JC (2017). Antegrade dissection and reentry as part of the hybrid chronic total occlusion revascularization strategy: a subanalysis of the RECHARGE Registry (Registry of CrossBoss and Hybrid Procedures in France, the Netherlands, Belgium and United Kingdom). Circ Cardiovasc Interv.

[12] Morino Y, Abe M, Morimoto T (2011). Predicting successful guidewire crossing through chronic total occlusion of native coronary lesions within 30 min. JACC Cardiovasc Interv.

[13] Guelker JE, Bansemir L, Ott R (2017). Validity of the J-CTO score and the CL-score for predicting successful CTO recanalization. Int J Cardiol.

[14] Wu EB, Brilakis ES, Lo S (2019). Advances in CrossBoss/Stingray use in antegrade dissection reentry from the Asia Pacific Chronic Total Occlusion Club. Catheter Cardiovasc Interv.

[15] Vo MN, Karmpaliotis D, Brilakis ES (2016). “Move the cap” technique for ambiguous or impenetrable proximal cap of coronary total occlusion. Catheter Cardiovasc Interv.

[16] Roy J, Hill J, Spratt JC (2018). The “side-BASE technique”: combined side branch anchor balloon and balloon assisted sub-intimal entry to resolve ambiguous proximal cap chronic total occlusions. Catheter Cardiovasc Interv.

[17] Azzalini L, Dautov R, Brilakis ES (2017). Procedural and longer-term outcomes of wire- versus device-based antegrade dissection and re-entry techniques for the percutaneous revascularization of coronary chronic total occlusions. Int J Cardiol.

[18] Maeremans J, Knaapen P, Stuijfzand WJ (2016). Antegrade wire escalation for chronic total occlusions in coronary arteries: simple algorithms as a key to success. J Cardiovasc Med.

[19] Spratt JC, Hanratty CG, Walsh SJ (2019). A guide to mastering antegrade CTO PCI. Part 1.

[20] Spratt JC, Hanratty CG, Walsh SJ (2019). A guide to mastering antegrade CTO PCI. Part 2.

[21] Godino C, Latib A, Economou FI (2012). Coronary chronic total occlusions: mid-term comparison of clinical outcome following the use of the guided-star technique and conventional anterograde approaches. Catheter Cardiovasc Interv.

[22] Carlino M, Azzalini L, Mitomo S (2018). Antegrade fenestration and re-entry: a new controlled subintimal technique for chronic total occlusion recanalization. Catheter Cardiovasc Interv.

[23] Colombo A, Mikhail GW, Michev I (2005). Treating chronic total occlusions using subintimal tracking and reentry: the STAR technique. Catheter Cardiovasc Interv.

[24] Brilakis ES, Mashayekhi K, Tsuchikane E (2019). Guiding principles for chronic total occlusion percutaneous coronary intervention: a global expert consensus document. Circulation.

[25] Carlino M, Godino C, Latib A (2008). Subintimal tracking and re-entry technique with contrast guidance: a safer approach. Catheter Cardiovasc Interv.

[26] Karacsonyi J, Tajti P, Rangan BV (2018). Randomized comparison of a CrossBoss First versus standard wire escalation strategy for crossing coronary chronic total occlusions: the CrossBoss First trial. JACC Cardiovasc Interv.

[27] Schumacher SP, Stuijfzand WJ, Driessen RS (2019). Impact of specific crossing techniques in chronic total occlusion percutaneous coronary intervention on recovery of absolute myocardial perfusion. Circ Cardiovasc Interv.

